# The quest for psychological symmetry through figural goodness, randomness, and complexity: A selective review

**DOI:** 10.1177/20416695241226545

**Published:** 2024-02-14

**Authors:** Daniel Fitousi, Daniel Algom

**Affiliations:** Department of Psychology, 42732 Ariel University, Israel; The School of Psychological Sciences, 26745 Tel-Aviv University, Israel; The School of Communications Disorders, Achva Academic College, Israel

**Keywords:** figural goodness, randomness, complexity, symmetry

## Abstract

Of the four interrelated concepts in the title, only symmetry has an exact mathematical definition. In mathematical development, symmetry is a graded variable—in marked contrast with the popular binary conception of symmetry in and out of the laboratory (i.e. an object is either symmetrical or nonsymmetrical). Because the notion does not have a direct graded perceptual counterpart (experimental participants are not asked about the amount of symmetry of an object), students of symmetry have taken various detours to characterize the perceptual effects of symmetry. Current approaches have been informed by information theory, mathematical group theory, randomness research, and complexity. Apart from reviewing the development of the main approaches, for the first time we calculated associations between figural goodness as measured in the Garner tradition and measures of algorithmic complexity and randomness developed in recent research. We offer novel ideas and analyses by way of integrating the various approaches.

Our goal in the current study is threefold. First and foremost, we wish to fill a major lacuna in the existing symmetry research. Wendell R. Garner's pioneering, foundational contributions (e.g. Garner, 1966, 1970, 1974) are all but ignored in large swaths of current symmetry studies. Garner and his work are not cited in Wagemans’ (1997) review or in that by Bertamini and Makin (2014) although the latter is processing and brain oriented. And the work is dispensed with a single sentence in the otherwise excellent recent review by Bertamini et al. (2018). Although Garner's work on figural goodness and symmetry is steeped in Gestalt psychology, it is not reviewed in Wagemans et al.'s (2012a, 2012b) comprehensive survey of Gestalt psychology. The authors discuss Garner's work on separability and integrality (Algom & Fitousi, 2016) but not Garner's relevant work on figural goodness and symmetry. The omission is particularly notable in their second review article (Wagemans et al., 2012b) devoted to explorations of the notion of holism. The concept of holism, one recalls, is a pillar of Garner's contributions to figural goodness and symmetry. And symmetry, in turn, is a signature of the presence of (one) object (e.g. Bertamini, 2006, 2010).

Of greater concern, when Garner's work is mentioned (Treder, 2010) and even examined (Van der Helm & Leeuwenberg, 1996), the discussion is incomplete (see Wagemans’, 1999, critique). Garner's theory is typically subsumed under the rubric of “transformational approach,” a grossly inadequate and eventually improper term. Garner surely employed transformations in his development, but they are tools (among others) in an evolved and complex system in which symmetry forms merely one area of application. The use of the term ignores the foundations of Garner's system in information theory (Shannon, 1948); as a result, key concepts like *uncertainty*, *information*, *set*, *inferred set*, or *redundancy* are overlooked. Given the common yardstick of information or uncertainty, Garner's ideas, including symmetry, enjoy broad generality and they apply across the senses. For a single illustration, Mach (1886/1959) thought that the sensation of symmetry is confined to vision (the almost exclusive focus on vision characterizes the great bulk of symmetry research to date). Mach even pondered if and how one-eyed people can possess a sense of symmetry and stated categorically that a sense of symmetry cannot be imagined in music. Garner, by contrast, demonstrated the presence of symmetry and other perceptual regularities in the auditory domain, too, given that his theory applies as naturally to temporal patterns as it does to spatial ones (e.g. Garner, 1970; Garner & Gottwald, 1968; Royer & Garner, 1970; see also Falk & Konold, 1997).

Our second goal was the attempt at streamlining the kaleidoscopic range of tools used in symmetry research by proposing a major distinction between studies that retain the flavor of the Gestalt principle of holism and studies that break the whole stimulus in various (mostly formal) ways. Considering the first class, “A Gestalt is an integrated, coherent structure or form, a whole” (Wagemans et al., 2012b, p. 1219), which is not determined by the individual component parts (Fitousi & Azizi, 2024; Koffka, 1935; Wertheimer, 1938; Wagemans et al., 2012b). Studying symmetry and figural goodness by preserving the whole stimulus is the hallmark of Garner's approach, fitting neatly into the original Gestalt teachings (e.g. Kimchi, 1992; Palmer, 1991). Research in the second class by contrast examines symmetry (and allied concepts) via the internal structure of the stimulus, ipso facto breaking down the whole stimulus onto its individual parts. The popular routine has been systematic numerical coding of the components. The resulting strings (often comprising arrays of 0 and 1) are subjected to advanced formal analyses informed by the notions of entropy, complexity, and randomness. Our distinction is surely not new; it emerges, often implicitly and under different terms, in portions of literature (e.g. Treder, 2010). Notably, the robust effort by Van der Helm and Leeuwenberg (1996) is driven by this distinction (already mentioned in their title). In the current assay, we use the two tracks of symmetry research as our guiding organizational principle.

Our review is by no means exhaustive. Within the framework of the whole-figure track, apart from Garner's seminal contributions, we discuss the development by Zabrodsky Hel-Or (1993). We do not review Wageman's Bootstrap Model (e.g. Wagemans, 1997; Wagemans et al., 1991), although the appealing simplicity of this processing model retains much of the flavor of Gestalt holism. We similarly do not discuss several developments by Palmer (e.g. Palmer, 1985, 1999; Palmer & Hemenway, 1978). Within the internal structure track, we review current models that approach figural goodness and symmetry by encoding the components of the spatial patterns in order to represent them by a sequence of symbols or strings. Apart from our own development, Fitousi strings, we discuss several models of algorithmic complexity, including the Lempel–Ziv system, the effort-to-compress (ETC) algorithm, and palindrome complexity in 1-D and 2-D. We also relate briefly to Atteave's (1954) conception of entropy and to Falk's (1975) applications on randomness.

A notable exclusion within this track is the Holographic Model (HM) of figural goodness developed by Van der Helm and Leeuwenberg (1996; see also Leeuwenberg & Van der Helm, 2013; Van der Helm, 2014). The HM is based on Leeuwenberg's (1969, 1971) structural information theory (SIT), which is, at its core, a coding procedure with the resulting sequences then reduced to maximum simplicity. Subsequently, the SIT was augmented mathematically by Van der Helm to encompass a large range of visual regularities. Put in very general terms, holographic regularity is present in a string if all the substructures entailed yield the same type of regularity (with various rules defining different kinds of regularity and visual results including symmetry). We appreciate the considerable quantitative prowess of the authors, but the omission of HM also reflects our lack of enthusiasm for this particular theory. We endorse the points made in Wagemans’ (1999) critique and can add our own, but our current assay cannot be technical. Wagemans’ verdict that “the many assumptions that must be made” render the HM “a less appealing… theory of how pattern goodness arises for human perceivers” (Wagemans, 1999, p. 612) reflects our own thinking.

Our third goal was a first attempt at associating measures from the two tracks. In particular, we calculated correlations between the Garner measures of figural goodness and current measures of complexity, patterness, and randomness. To anticipate, we did record the presence of associations in the face of constraints imposed by the specific nature of each measure. Uncovering the various routes to symmetry remains a daunting task.

Finally, we should mention our exclusion criteria. There are several (recall, the current review is selective with special focus on the foundation of symmetry). We limit our discussion to figural goodness and symmetry and do not consider other species of visual regularities, notably repetition or translation (there are multiple names for each type of regularity including symmetry). Examination of the neural and biological substrates of symmetry processing similarly falls outside of the current purview (for expert reviews of this subject see, Bertamini et al., 2018; Bertamini & Makin, 2014; Cattaneo, 2017; Treder, 2010). Pursuant to the previous point, we do not discuss RT studies of symmetry detection (e.g. Baylis & Driver, 2001, 1995; Bertamini et al., 1997; Cattaneo, 2017). This probably reflects our own bias. Our approach is foundational, exploring the essence of the notion of symmetry. The contrast can perhaps be illustrated by considering the study by Baylis and Driver (1995). In this investigation (as well as in a series of studies by these authors), the participants made speeded judgments of symmetry, but not a word is spent in the report on the definition or nature of symmetry (for the reader and the participants alike). Consider by way of contrast a circle, the kind of stimuli considered in the present article; this shape is infinitely symmetrical, but it seems meaningless to ask about symmetry or to detect the presence of symmetry thereof. Again, our approach seeks to uncover the roots of the concept of symmetry. Given the focus on simple stimuli, we also do not discuss the human face (but see Fitousi, 2024). Lastly, a further topic left undiscussed is pattern attractiveness (e.g. Bertamini et al., 2013), although ratings of figural goodness might be considered close.

The organization of the text is as follows. A historical perspective is followed by the major distinction guiding the presentation: whole figure analysis versus internal structure analysis in that order. In the Conclusion we allude to further topics aligned with symmetry.

## Symmetry, Gestalt, and Information: A Bit of History

Symmetry has its roots in antiquity. The Greek word meant balance and harmony in figures, sculptures, and particularly in architecture. Symmetric works were judged to be pleasing to the senses to the extent that the equation, symmetric = beautiful, held throughout the Greco-Roman world (Weyl, 1952). After a long and convoluted history through the Middle Ages and the Renaissance (Weyl, 1952), Gestalt psychologists invented the notion of “figural goodness” early in the 20th century, retaining the flavor of the original Greek notion (Wertheimer, 1912; Koffka, 1922; see also, Kimchi, 1992; Mach, 1886; Wagemans et al., 2012a, 2012b). Good figures are experienced as simple, regular, orderly, and predictable. To account for these *subjective* sensations, the Gestaltists suggested objective features such as redundancy, complexity, or symmetry. Notably, all measures were “holistic” in that they referred to the whole figure. To the Gestaltists, “perception… shows a character of totality, a form, a *Gestalt*, which in the very attempt at analysis is destroyed” (Heidbreder, 1933, p. 331). People see unified wholes, trees, clouds, rectangles, or circles, rather than assemblages of elements. The problem was that the objective determinants of figural goodness identified by the Gestalt theorists lacked a rigorous quantitative definition. No further progress was made until concepts from the mathematical information theory became available to psychologists.

The breakthrough came with Claude Shannon's publication of his two-part paper on a mathematical theory of communication (Shannon, 1948). Let *x_i_* be an event from a set of *N* alternative events with probability of *p*(*x_i_*). Following Shannon's development, the information *I* carried by the occurrence of *x_i_* can be written as

(1) $$I(x_{i})=-\log\;p(x_{i})\;\;\mathrm{in}\;\;\mathrm{bits},$$

with the bit, the unit of information, signaling that the base of the logarithm is 2. If each alternative event in the set has the same probability of occurrence, one obtains

(2) I(x)=logN

The terms information *I* and uncertainty *U* are often interchangeable because the amount of information gained is the amount of uncertainty or entropy reduced. So, using Equation 1, the weighted average uncertainty of a set is

(3) U(x)=∑p(x)logp(x)

Equations 1–3 apply readily to univariate uncertainty distributions, but it is possible to define a given probability distribution in terms of more than one dimension or variable. It is important to recognize that there is no real difference between a distribution defined in terms of a single variable and one defined in terms of two or multiple variables. In all cases one applies the same basic statistics to various classifications of the stimuli. The information measure is nonmetric, so it is not affected by the classification of the stimuli. This truism granted, the bivariate case supplies further useful measures, including those of information transmission, contingent and conditional uncertainty, and channel capacity (Fitousi, 2013, 2023; Garner, 1962; Miller, 1956). These, in turn, clarify the relation between the notions of redundancy and correlation.

It is difficult to overstate the impact of Shannon's development on psychology. Information theory opened the possibility of quantification of previously undefined terms and ideas. For example, Gestalt theorists discussed (vertical) bilateral symmetry (the only type considered; Wagemans, 1997; see also Mach, 1886), but they did not quantify the notion nor did they believe it to be the sole determinant of figural goodness. With Shannon's measures kept firmly in their toolkit, researchers were quick to explain pattern goodness, indeed patterness, in terms of information (e.g. Attneave, 1954, 1959; Hochberg & McAlister, 1953; Hyman, 1953). The central idea was that patterned sequences and good figures contained less information due to their makeup that entails order and redundancy. Thus conceived (or constructed), good figures seem uncomplicated and well organized to the observer, easier to remember and discriminate than poor figures (e.g. Bertamini et al., 2018). Applying Shannon's (1948) tools, “Good figures were shown to be objectively simpler than bad ones in a well-defined informational sense” (Palmer, 1991, p. 25).

The value of these pioneering contributions granted, these early forays into information theory have fairly rapidly reached an impasse. The reason was that the mode of research and explanation employed by theorists like Attneave and Hochberg violated the basic tenet of Gestalt psychology on the totality of the whole figure (Heidbreder, 1933; Wertheimer, 1938). These theorists sought to explain the Gestalt concept of figural goodness and patterness but, contra Gestalt, their informational analysis entailed breaking down the stimulus into local components. The style of analysis espoused by these theorists did not fit well with Shannon's formulation, too. Considering Equations 1–3, the most natural application is to construe event x_i_ as a whole stimulus, albeit a member of a set. However, Attneave, Hochberg, and their colleagues eschewed the notion of set; they focused on the stimulus, a single stimulus at that. Attneave (1959) acknowledged this feature of his approach for example when discussing the term “surprisal value” or information value of a single event, to wit, “surprisal… is useful because it refers to a *particular event*…, not to a whole range of alternatives as is usually the case with *information*, and necessarily the case with *uncertainty* (pp. 6–7, first emphasis added). However, the notion of set (N in Equation 2) is vital to Shannon's development.

It took the genius of Wendell R. Garner to incorporate the notion of set into psychology in a way that is also commensurate with the Gestalt style of explanation (Garner, 1966, 1970, 1974). However, the trail blazed by Attneave, Hochberg, and their colleagues has recently gained traction. This research concerns the internal structure of the stimulus. In addition to Attneave's (1959) analysis of entropy of sequences, recent research often focuses on the concept of randomness (the inverse of patterness or figureness). The relevant notions include terms such as *subsymmetries*, *palindromes* (an ancient term just a bit younger than symmetry), and mainly *complexity*. Therefore, it is possible to classify the psychological treatment of symmetry into two classes: that which considers the stimulus as a whole and that which considers the internal structure of the stimulus. A similar though not fully overlapping distinction is implicit in portions of the literature, for example, that drawn between transformational and holographic classes of theories by Van der Helm and Leeuwenberg (1996). We discuss both tracks, supporting our discussion with novel analyses.

## Psychology of Symmetry: The Whole Stimulus Track

### Garner Sets and Mathematical Groups

For Garner, the main takeaway from the information theory was that perception of a stimulus is always affected by alternative stimuli—those stimuli that could have been present(ed) but were not present(ed) on that particular occasion (trial). Looked at from an informational perspective, there is no such thing as perception of a single stimulus onto itself. The properties of the single stimulus are simultaneously the properties of the set of all alternative stimuli within which it exists. But how can one find that set? In some cases, life experience provides a ready clue as, for example, when the symbol *e* is presented. People probably perceive the letters of the alphabet as the relevant set. With most other stimuli, Garner's idea was subjecting the figure to certain spatial transformations by way of producing the set of alternatives. The key observation was that good figures produce a smaller set of alternatives than poor figures. Although he did not refer to the mathematical group theory (or indeed to symmetry), Garner's development is stunningly similar to the fundamental notion of symmetry in group theory, to wit, “We say something is symmetrical when it looks the same from more than one point of view” (Carter, 2009, p. 25).

**Figure 1.**
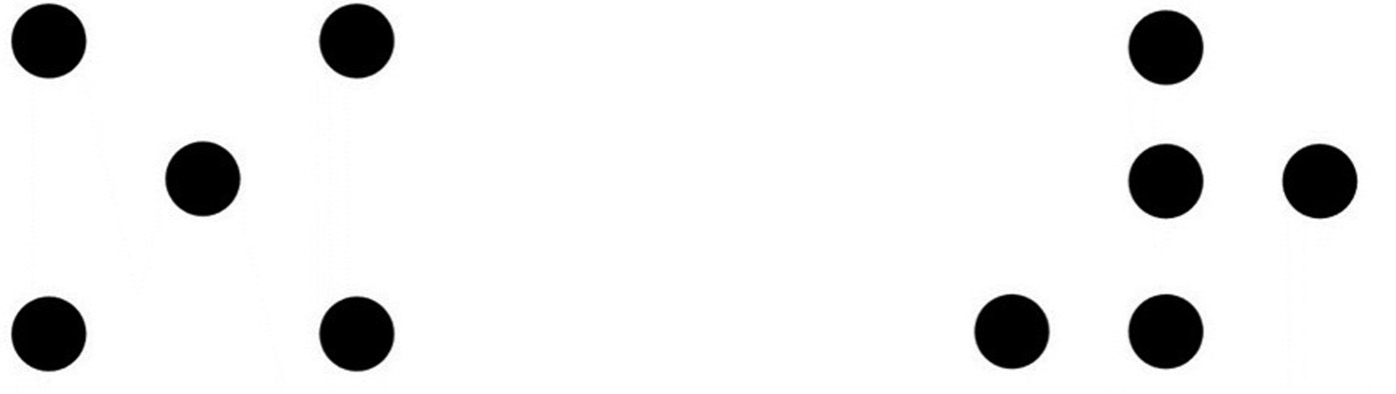
Two dot patterns defined by their R & R subsets and by their similarity groups. Left panel: Size of the R & R subset = 1; Similarity group = 8 [I, V, H, L, R, 90°, 180°, and 270° rotations]. Right panel: Size of the R & R subset = 8; Similarity group = 1 [I]. I: identity; V: vertical reflection; H: horizontal reflection; L: left-diagonal reflection; R: right-diagonal reflection. Based on Palmer (1991).

In a seminal experiment, Garner and Clement (1963) placed five dots in an imaginary matrix of 3 × 3, creating a set of 90 such stimuli (Figure 1). In one task, each pattern was judged for figural goodness, whereas in another task the 90 stimuli were sorted freely into subsets based on similarity; the subsets did not have to be of the same size. Startingly, the correlation between these two behavioral measures—judged goodness of the figure and the size of the subset created that contained the figure—amounted to .84. Clearly, good patterns were perceived as members of small subsets and poor patterns as members of large subsets. This subjective perceptual result can be captured in a powerful manner by the *objective* operation of spatial transformations applied to each pattern. Garner and Clement (1963) used four central rotations by steps of 90° and four central reflections around the vertical, horizontal, the left, and the right diagonal. The different patterns per stimulus generated by Rotation and Reflection define its *R & R subset*. It was then demonstrated that good patterns had small *R & R subsets*, whereas those rated poor had large *R & R subsets*. The pattern at the left in Figure 1 looks the same throughout all the spatial transformation, whereas that at the right looks different with each transformation—the result epitomized in the title of Garner's (1970) work, “Good Patterns Have Few Alternatives.” It is also important to recognize that the participants rated each pattern *singly* and were never shown the other patterns in the *R & R* subset. Consequently, Garner and Clement (1963) termed the patterns produced by the spatial transformations as *inferred* subsets and suggested that figural goodness is an inverse function of the size of its *inferred R & R subset*.

One notes the close association of Garner's theory with Shannon's development, on the one hand, and with the Gestalt viewpoint, on the other hand. Concerning information theory, Garner conceived the notion of set (Equations 1–3) as a *psychological* concept, which, in turn, enabled application to perception of the variable of *redundancy* (the inverse of uncertainty or information). Good patterns, in contrast to poor patterns, are predictable since they remain the same across reflections and rotations (hence they reside in small *R & R subsets*). In Garner's (1970, p. 42) formulation, “Poor patterns are those which are not redundant and thus have many alternatives, good patterns are those which are redundant and thus have few alternatives, and the very best patterns are those which are unique, having no perceptual alternatives.” Concerning Gestalt theory, Garner's analysis is particularly appealing because it applies to whole figures. As Palmer (1991, p. 25) notes with respect to Garner's analysis, “There is no sense in which the patterns need to be broken into piecewise components to apply the analysis.”

The mathematical analysis of symmetry provides an alternative account for pattern goodness. In the most general sense, an object is symmetrical with respect to an operation if that operation preserves the property of interest in the object. Getting closer to the present case (and ignoring several strictures), the operation can be spatial transformations such as flipping the figure around certain axes and rotating it along certain angles. Those transformations under which the figure remains invariant specify its symmetry group. Thus, it becomes evident that the symmetry group of a circle is infinitely large or that the symmetries of the square are more numerous than those of the rectangle. It is also easy to see that good figures have more symmetries than poor figures. With the Garnerian transformations in force, the good pattern at the left of Figure 1 has eight transformations in its symmetry group, whereas the poor pattern at the right has just a single transformation (identity) under which it is invariant. In fact, as Palmer (1991, 1983) observed, symmetry group analysis is isomorphic to Garner's R & R set theory; the respective sizes are inversely related to one another (see e.g. Royer, 1981). Garner's formalism focuses on *sets* of patterns, whereas group theory focuses on *transformations* of a pattern. Employing the latter, one can say that good figures have more symmetries than poor figures.

**Figure 2.**
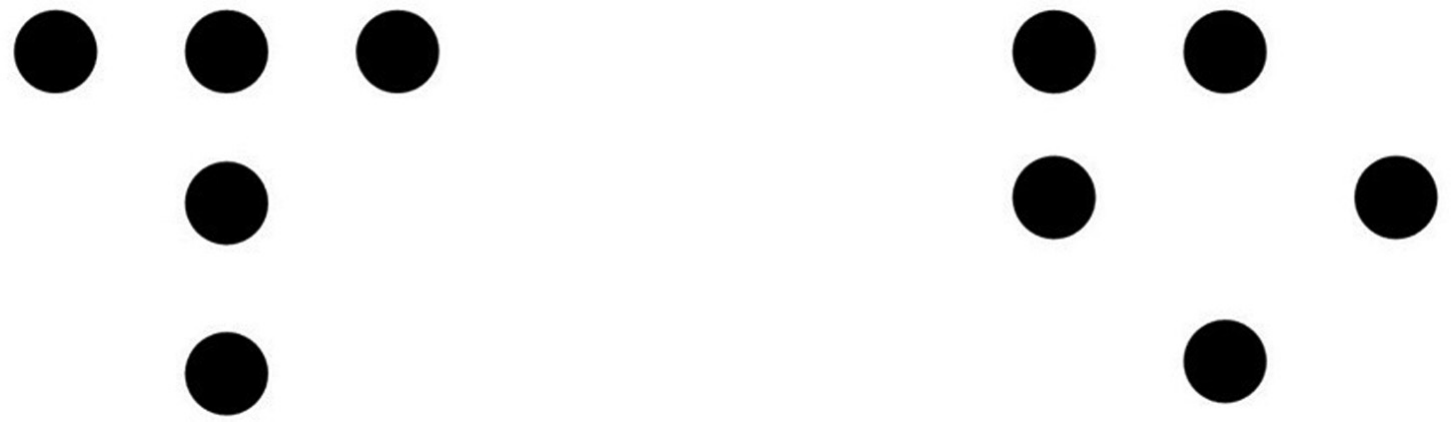
Two dot patterns with the same size (4) of R & R subsets but produced by different transformations. Left panel: The relevant subset is produced by identity and the vertical reflection. Right panel: The relevant subset is produced by identity and the left-diagonal reflection. Based on Palmer (1991).

Despite the coequality, there are advantages in using each system. An important advantage of group theory is that different figures and their attendant symmetry groups can be compared with respect to the type of transformations in addition to their number (Palmer, 1991). The two patterns in Figure 2 have *R & R subsets* of the same size, yet group theory identifies the different transformations yielding those equivalent subsets. Garner's theory does not disambiguate such patterns. However, different transformations can carry distinctive psychological effects. For example, vertical bilaterally symmetrical patterns are consequential to perception more than horizontally symmetrical patterns (e.g. Bertamini et al., 2018; Mach, 1886), and only the former symmetry was considered by the Gestalt theorists. On the other hand, the connection with information theory and particularly with notion of redundancy is salient in Garner's development. Recall that, cognitively, a stimulus is always a member of a collection of meaningfully related alternative stimuli. This collection is a subset of the total set of stimuli created by the given stimulus dimensions; it is the *inferred* subset. Finally, and most important, Garner (1966, 1970, 1974) has shown that any subset drawn from the total set (preserving the stimulus dimensions) contains redundancy and that the smaller the size of the subset, the greater the redundancy. Good figures exist in small subsets; hence they are redundant. Garner used the tools of information theory to quantify these notions. As a result, one can say that good patterns convey little information and that poor patterns are informative.

For a final observation, note that the group theory is appealing from a Gestalt point of vantage as much as the Garner's theory. Group theory also works with “objects” or with whole figures.

### Zabrodsky Hel-Or's Symmetry Distance

In 1993, Hagit Zabrodsky Hel-Or advocated a *continuous* measure of symmetry (Zabrodsky, 1993; Zabrodsky et al., 1993). In her development, the symmetry distance (SD) of a figure is defined as the minimum mean squared distance required to move points of the figure in order to obtain a symmetrical figure. The measure of the SD can be applied to all types of symmetry in any dimension. Applying the SD enables one to compare any two figures in terms of their amount of symmetry and to assess the amount of the various symmetries (e.g. vertical, horizontal, rotational) in a single figure. Therefore, the intuitive yet vague impressions made by figures with respect to their goodness or symmetry can be quantified by a repertoire of the various “amounts” of symmetry. Our depiction here cannot be technical other than to note again that Zabrodsky Hel-Or's development is rigorous mathematically and sustained by proofs and by a geometric algorithm to evaluate the symmetry transform. Apart from the formal evaluation, the success of transforming the figure onto a symmetrical figure (for any specified type of symmetry) can also be appreciated visually by the observer.

How does Zabrodsky Hel-Or's measure of continuous symmetry relate to human perception of figural goodness? To address this question, Zabrodsky and Algom (1994) first applied Zabrodsky Hel-Or's SD measure to Garner's dot patterns. The results are summarized in Table 1. First, note the rich yield of symmetry measures for each dot pattern. Second, the best Garner pattern (size of R & R subset = 1) has a perfect symmetry. Third, the SD symmetries portray an inconclusive picture with respect to the poorer patterns (subset sizes of four and eight, respectively). One must conclude that figural goodness as defined by Garner and figural goodness as given by continuous symmetry are only moderately associated.

**Table 1.** Analysis of Garner Dot Patterns by Continuous Symmetries (Exemplars Indicated in Parentheses).

Subset size			
	1 (left pattern in Figure 1)	4 (patterns in Figure 2)	8 (right pattern in Figure 1)
Continuous symmetry via symmetry distance			
v	0	0	6.10
h	0	12.24	9.76
hv	0	6.12	4.88
vh	0	6.12	4.88
C_2_	0	12.24	3.66
C_4_	0	12.24	6.71

Note: v: vertical mirror symmetry; h: horizontal mirror symmetry; hv: left-diagonal mirror symmetry; vh: right-diagonal mirror symmetry; C_2_: rotational symmetry of order two (120°); C_4_: rotational symmetry of order four (90°).

Next, Zabrodsky and Algom (1994) attempted to examine more directly the relation between the SD measure of continuous symmetry and human perception. Is the former development of symmetry commensurate with human experience of figural goodness? To test the presence of a contingency, the authors created sets of random shapes such that, within a set, each shape had a different value of SD with respect to the mirror- and rotation-symmetry (of order two). Judgments of figural goodness were obtained for each shape. The results revealed moderate correlations between the subjective figural goodness and the SD measure in both symmetry subgroups of reflection and of rotation. However, as is the case in Table 1, it was the highly symmetric figures that have mainly driven the correlations.

For a final important point, the symmetry development by Zabrodsky Hel-Or also works on whole figures.

## Psychology of Symmetry: The Internal Structure Track

The value of the pioneering contributions by the Gestaltists and the commensurate developments by Garner and Palmer granted, another track—fine-grained analysis of stimuli—has contributed to the vitality of the modern study of symmetry. Recent research has shown how local elements and operations performed on them can affect perceived patterness, symmetry, or figural goodness. Indeed, there are good reasons for considering local analysis. First, physiology favors a local analysis; the visual system is rather limited in its ability to process whole figures because it relies on information coming from small receptive fields that cover segments of spatial locations or objects (Marr, 1982). Another implication of physiology is that at times segments may be processed serially. Palmer (1991) and Weber (1993) investigated space–time symmetries in vision, that is, symmetry of patterns that vary in time as well as in space. They found comparable results between space–time and space–space symmetries. Finally, global processing often is unrealistic in a cluttered environment where objects can be occluded. Thus, the analytic venue pursued in the early days of cognitive psychology is enjoying a revival, sustained by recent advances in computer science and mathematics. We next review portions of that development, contributing several results of our own.

### From Garner's Dots to Fitousi's Strings to Algorithmic Complexity

**Figure 3.**
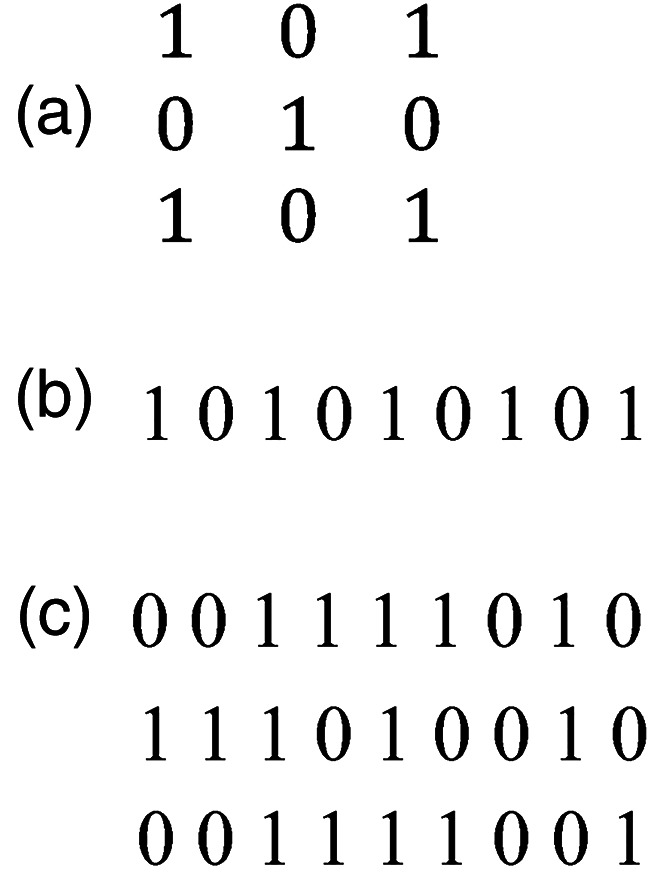
(a) The good pattern from the left of Figure 1 with the numerals 1 and 0 replacing the dots and empty cells, respectively. (b) The resulting one-dimensional string for this pattern; this same string results through all eight ways of mapping. (c) Three of the eight different strings produced by the poor pattern at the right of Figure 1. For example, the string on top is produced with coding by columns from left to right then proceeding within a column from the top.

One of us, Daniel Fitousi, suggested the following string representation of the Garner dot patterns for analysis. For any given pattern within the matrix of 3 × 3, let the numeral 1 stand for a dot and the numeral 0 for an empty cell. Then, one can encode the pattern by its rows, starting from the top or from the bottom; within a row one can proceed from the right or from the left end. Alternatively, one can elect to encode by columns, starting from the left or from the right; within a column, one can proceed from top or from the bottom end. So, there are eight modes of coding in all. To illustrate, consider the pattern at the left of Figure 1—one of the very best patterns. Replacing the dots and the empty cells with the numerals 1 and 0, respectively, gives the matrix of Figure 3a. The associated string is shown in Figure 3b. By which coding? By *all*: The same single string results regardless of which one of the eight possible ways of coding is used (the reader is encouraged to verify this quite effortlessly). Good patterns produce the same string or very few different strings when they are subjected to those eight modes of binary coding. Such a result is precisely what we would expect if we considered coding as a proxy for human scanning of a pattern. Good patterns remain invariant when looked at from different angles. Poor patterns, in contrast, look unalike in appearance when looked at from different angles, producing a different string with each coding. Three of the eight different strings for the poor pattern at the right of Figure 1 is shown for illustration in Figure 3c.

We are not the first to use symbolic encoding of stimuli of course. For example, Attneave (1959) used binary encoding via sequences of ones and zeros to specify the location of a particular square on an otherwise empty 8 × 8 board. Attneave (1959) provided the logic and computational routine to measure the entropy of any ordered binary sequence (easily extended to other sequences). Subsequently, Falk (1975; see also Falk & Konold, 1997) applied Attneave's analysis to grids of black and white squares. The stimuli comprised 10 × 10 cells, and Falk used Attneave's procedure to quantify the grids’ *objective* randomness. We, too, make use of Attneave's tools. However, Fitousi's strings are the first attempt to encode Garner's dot patterns with the predominant purpose of *representing* them. Consequently, the *number*, *identity*, and *order* of the strings (per pattern) are as important as their entropy. The close figure–string affinity also means that what is true of the sequence translates directly to the relevant pattern (and vice versa).

Given Garner's rotation and reflection operations with respect to the 3 × 3 matrix and our coding of the same matrix by triplets (of its rows or columns), the close association between the size of the R & R subsets and the associated strings comes as little surprise. Thus, the size of the R & R subset for a pattern is equal to the number of unique strings produced by that pattern (e.g. one for the best pattern, eight for the poorest pattern). By group theory, the number of *same* strings per pattern reflects the number of transformations that leaves that pattern invariant (e.g. eight for the good pattern, one (identity) for the poor pattern). And, similarly to the group theory, the *type* and *order* of the unique strings disambiguate patterns belonging to R & R subsets of the same size (see e.g. the pair of patterns in Figure 2).

However, the main value of Fitousi's strings lies in forming the crucial bridge between randomness and pattern goodness. The affinity between the two has been recognized of course (e.g. Falk, 1975; Falk & Konold, 1997; Garner, 1970; Griffiths & Tenenbaum, 2003; Lopes & Oden, 1987; see also Garner, 1974) but Fitousi's strings hold the promise of aligning the respective bodies of work in a more detailed manner.

The key concept in contemporary randomness research is that of complexity with the pertinent tools lending themselves most naturally to sequences of symbols, hence the power of the Fitousi strings. The fundamental idea is that the complexity of a string is based on its minimal description. This idea is realized in a variety of computer languages that quantify complexity or randomness (hence also patterness and symmetry). The programs go by a variety of names, including algorithmic information, algorithmic complexity, algorithmic randomness, or Kolgomorov complexity but share the minimal description principle (e.g. Falk & Konold, 1997, p. 306): “The algorithmic randomness of a binary-digit sequence is the bit-length of the shortest computer program that can reproduce the sequence.” This definition of randomness and its inverse, patterness, carry considerable appeal. Sequences marked by simple patterns and symmetry are easy to describe, whereas random sequences are indescribable.

**Figure 4.**
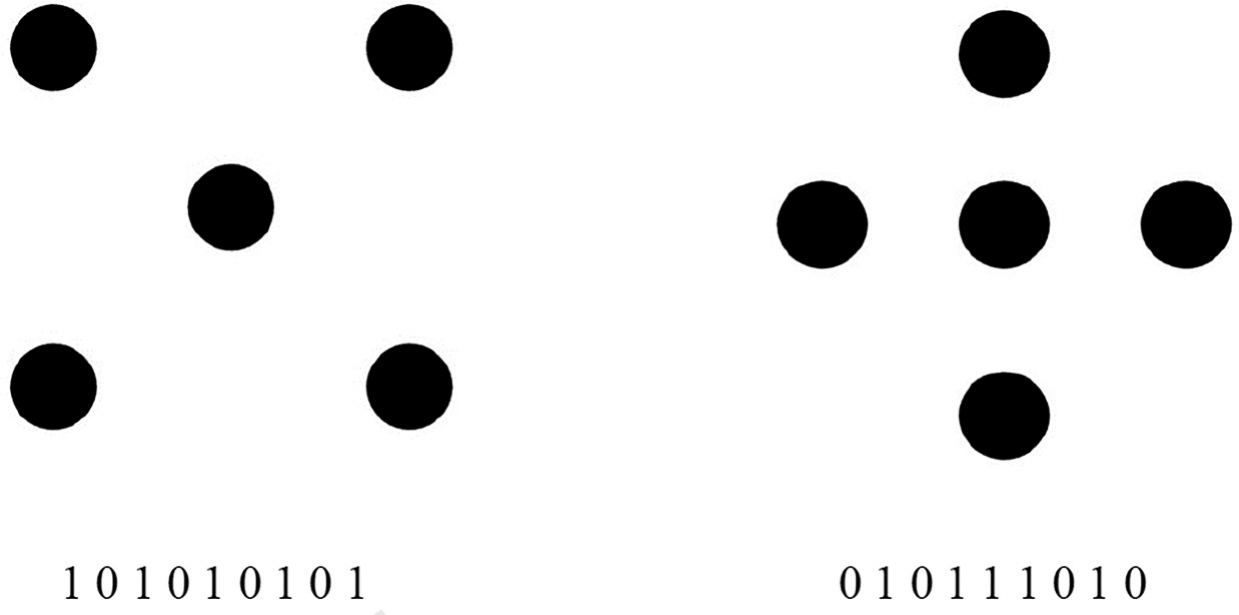
The pair of Garner's best dot patterns marked both by the minimal R & R set of 1. Each pattern remains the same under all transformations, so that they have no alternatives. Nevertheless, the sequence below each figure reveals that the figure at the left is better than the figure at the right.

Consider for example the pair of sequences shown in Figure 3 (panels b and c, top). The former sequence, representing the best dot pattern, can be described concisely by “start with 1, then alternate to the end.” The latter, representing a poor dot pattern, defies such description. Fitousi's strings can disambiguate even the best dot patterns, considered equivalent to date (see Figure 4). The two patterns in Figure 4 are equivalent in terms of R & R subset size (1) and in terms of symmetry transformations that leave each invariant (eight), but *not* in terms of Fitousi strings. As the strings below each figure reveal, the sequence for the figure on the left can be described more concisely than that for the figure at the right; the former is more redundant or less complex. Consequently, one can say that Garner's pair of best figures differs in figural goodness. The figure on the left was judged only marginally better by the observers in the Garner and Clement (1963) study; however, in the same study the figure on the left had an appreciably smaller size of the psychologically inferred set of similar patterns. By this powerful measure one can maintain that the figure on the left is a better figure than the figure at the right (Is that the reason for including the left-hand figure to represent five in the game of domino?).

A glimpse at the string representing the pattern at the *right* in Figure 4 reveals that it is still mirror-symmetric, which means that it does not matter whether one reads it from left-to-right or from right-to-left. This is also true of the parent figure itself of course, which accounts for its goodness and superiority over all other patterns except for the other member in the R &R subset. Attneave (1959) provided a routine for computing the redundancy in binary sequences based on an analysis of all ordered pairs in the sequence. His measure captures the new information provided by the second member of the pair. Because each member in a sequence (except the first) is the second member of some pair, Attneave's entropy is, viewed generally, the new information added by each digit in the sequence. Obviously, the simple alteration of the sequence at the left of Figure 4 is completely redundant: Every symbol is fully predicted by its immediate predecessors, so that no new information is provided by any and all symbols in the sequence. This sequence and the pertinent pattern have zero or minimal second-order entropy (subject to the proviso that the symbols of one and zero are not equiprobable). Subjecting the string at the right of Figure 4 to Attneave's analysis reveals that it is a bit less redundant than the zero-redundant string at the left (there is no need to specify this trivially obvious difference). The symbols at the right are lawful and regular to be sure but not completely so.

Atteneave's (1959) computation of entropy in a binary sequence is simplest when considering the pairs included (called “digrams”); when a perfect prediction of a symbol is possible if the immediately preceding symbol is known, the sequence has second-order redundancy (as does the sequence at the left of Figure 4). At the other end, second-order redundancy is minimal—and entropy and randomness are maximal—when all four digrams are equiprobable (which is not the case with each of the sequences of Figure 4) and when no dependencies exist between successive symbols. The sequence at the right of Figure 4 thus falls between these two extremes, although it is much closer to being redundant; it contains little entropy and is definitely not random.

Therefore, symmetry can be applied locally to Attneave's (1959) digrams or pairs of digrams. Second-order redundancy means that all pairs of digrams, indeed all digrams, are symmetric and predictable. Maximal second-order redundancy is tantamount to maximal symmetry, so that the two notions are virtually isomorphic (akin to the notion of subsymmetry discussed below). To recap, good figures and sequences (a) are amenable to short efficient descriptions, hence are not complex, (b) are redundant, hence noninformative, and (c) are symmetrical. These properties are interconnected if not interchangeable. For example, it is symmetry that guarantees compression into short descriptions, which, in turn, enables nameability. These properties explain why good figures, indeed patterned stimuli in general, are easy to name, learn, memorize, or discriminate. With complex sequences all of these tasks are more difficult. And completely random, maximum-entropy or maximally complex sequences are incompressible. How does one convey random blobs on a canvass when the stimulus lacks a name and defies (covert) verbalization? The only way to convey a random stimulus is by copying or reproducing it.

Finally, Falk and Konold (1997) attempted to identify the psychological mechanism accounting for the *subjective* experience of apparent randomness. Of several candidate mechanisms, the data supported that of *the difficulty of encoding*. People judge randomness, and by implication patterness, by the difficulty experienced of (tacitly) encoding the stimulus. The subjective variable of difficulty of encoding agrees well with the objective variable of difficulty of compression. In the context of Garner's dot patterns, good patterns were rated low on perceived complexity, and they were ascribed shorter verbal responses (Garner & Clement, 1963; Hamada & Ishihara, 1988).

### Associating Figural Goodness and Complexity

These analyses comprise our novel contributions. Here, too, our point of departure is the stimuli and results reported in the archetypal study of Garner and Clement (1963). As we recounted, Garner and Clement (1963) presented 90 different dot patterns—generated via rotations (by 90°) and reflections of 17 root patterns (Garner & Clement, 1963; Weyl, 1952). Applying our encoding-by-triplets procedure resulted in 90 Fitousi strings, which can be mapped back to the 90 patterns presented by Garner and Clement. Such strings served as the platform enabling the pioneering application of algorithmic complexity to the Garner patterns. We related complexity to three variables entailed in the Garner and Clement (1963) study, one objective and two subjective. The *objective* variable is the size of the R & R subset of which the pattern is a member. The first *subjective* variable is the size of the collection of patterns, grouped together by the observers on the basis of similarity that the pattern belongs in. The second *subjective* variable is simply that of judgments of figural goodness.

Several caveats seem in order before proceeding. Our idea of associating Garner's figural goodness and latter-day complexity is a bold one and beset with problems. First, modern measures of complexity were developed to account for randomness. It is by extension that we apply them to the inverse of randomness, patterness, and to pattern goodness at a stretch. Second, complexity algorithms work best with long sequences, whereas those associated with Garner's simple figures are very short. Third, the Garner variables are also constrained by range (e.g. the objective variable of the R & R set size has merely three values). Consequently, our results are best considered in an ordinal sense as a first foray into this realm. The very presence of an association would be significant and interpretative.

As we recounted, the measure of *Kolmogorov complexity* (Kolmogorov, 1963, 1965) defines the length of the shortest computer program that generates the string. Because the Kolmogorov complexity is not computable, lossless compression algorithms have been employed to approximate an upper bound on the degree of complexity. We applied the Lempel–Ziv algorithm, defined as the number of different substrings encountered as the string is viewed from beginning to the end by the computer program (Lempel & Ziv, 1976; Ziv & Lempel, 1977).

#### The Lempel–Ziv Algorithm Applied to Garner Stimuli

The Lempel*–*Ziv (hence LZ) algorithm compresses a string by creating a code word for every new substring and then using this code instead of the substring when it meets it again. The fact that in the LZ, information is transmitted bit-by-bit, whereas in the Garner dot patterns, the spatial elements are presented in parallel does not pose a problem. Any spatial pattern that is constructed according to a predefined alphabet (e.g. sequences of zeros and ones) can be coded into a temporal pattern and transmitted through a serial channel (e.g. Morse code). The informational regularities that characterize temporal transmission can be decoded back to a spatial representation. Obviously, in the brain, patterns are represented by temporal coding of neuron firings (Freiwald et al., 2009), which retain their informational content and allow for decoding them back to spatial codes. The LZ algorithm has been used extensively in coding two-dimensional images into GIF files, which are unidimensional strings of zeros and ones.

First, we wished to relate the two *objective* measures of randomness or patterness calculated with respect to the same stimuli, its size of the R&R subset, and its value of LZ complexity. For this analysis, we considered the 17 root patterns and encoded each pattern in all eight possible ways (17 × 8 = 136). This complete set includes 46 identical strings or identical figures issuing from rotations and reflections of the members of the root set (see Weyl, 1952). Note that in their study Garner and Clement (1963) presented only the sample of 90 different figures.

**Figure 5.**
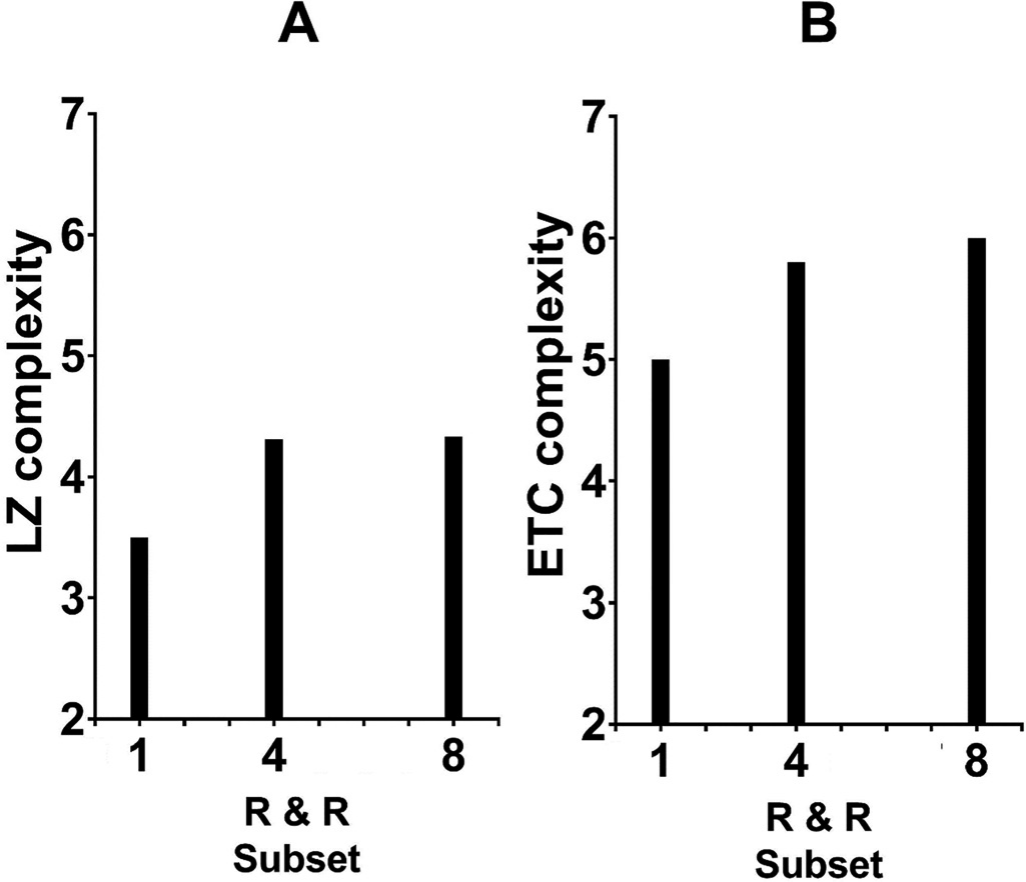
Panel a: Average LZ complexity as a function of the R & R subset. Panel b: Average ETC complexity as a function of the R & R subset. The variable at the abscissa is the size of the subset of the dot patterns that can be created from rotation and reflection (R&R) of each of 136 patterns issuing from 17 basic dot patterns. The dot pattern stimuli used by Garner can result in three values of the R&R subset size. Each individual pattern can be decoded into a string of 1 and 0 in eight different ways of reading the spatial pattern (Fitousi strings). The variables on the ordinate are the Lempel–Ziv complexity applied to each string (left) and the Effort-to-Compress complexity applied to each string (right).

For each of the 136 strings, we computed its LZ complexity. In the next step, we calculated the average complexity of the patterns belonging in each of the three R & R subsets. The results are shown in the left panel of Figure 5. Clearly, there is an association, although, quite expectedly, it is not a strong one numerically. For one, the Garner variable of R & R set size is restricted to a mere three values. For another, the LZ complexity is not zero (nor even one) for finite strings, including those comprising the same symbol. For example, the LZ algorithmic complexity is 2 for such a simple string as 00000000 (the reason is that there is still a need for a pair of instructions: the string is described by the symbol of 0 and then the program should repeat it eight times). This explains why the LZ complexity for the best dot pattern (presented in Figure 4) is already 3 and the average complexity of the two best patterns is larger than 3 (mean of 3.25). It is likely due to these limitations that the observed association is weak.

Next, we wished to relate the objective variable of algorithmic complexity to the *subjective* variables of (a) size of the collection of patterns classified by the observers to be similar to the tested pattern and (b) the judged figural goodness of the pattern. Although both correlations were significant statistically, the values were rather small. The LZ complexity-group size correlation was .21; given that poor patterns exist in large collections, this means that poor patterns tended to entail greater complexity. The correlation between the LZ complexity and the experience of figural goodness was −.18. This means that the better the figure is perceived subjectively, the lower its complexity tends to be objectively.

#### The Effort-to-Compress Algorithm Applied to Garner Stimuli

The second species of complexity that we employed in the present contribution was the Effort-to-Compress (ETC, Nagaraj, Balasubramanian, & Dey, 2013). On each iteration, the ETC algorithm identifies the input sequence that is most frequent and replaces it with a new symbol. The program stops when the string of symbols becomes constant. The ETC complexity is defined by the number of iterations required for reducing the input string into 1 or to a constant string. This definition of complexity bears affinity to Leeuwenberg's (1969, p.10) idea of coding figures and sequences: “The information of a figure [is] the number of operations … which must be successively applied in order to rob the given figure of its content.” Thus the ETC complexity of a sequence consisting of the same single symbol (e.g. AAAAA) is 0. The ETC complexity of the sequence standing for the best Garner dot pattern (presented in Figure 4) is 4—four iterations are required to reduce it to the minimal length of 1. Consider just the first iteration by which 101010101 reduces to 22221 (because 10 appears most frequently). Note incidentally that shortening that sequence by deleting its last member (hence no longer a Garner string) has an ETC complexity of 2.

We followed the same routine with ETC that we did with LZ. The results of relating the objective measures of the R &R set size and the ETC complexity (performed on the same stimulus) appear at the right-hand panel of Figure 5. Clearly, there is an association between these two measures of sequence structure with the order of complexity commensurate with that of the R & R set size, including the larger values. Thus, the mean complexity for the set size of 4 was 5.81 and for the set size of 8 was 6.

We next related ETC complexity to each of the pair of subjective variables from Garner and Clement's (1963) study. The correlations were significant statistically but with low values. The correlation between the ETC and the width of the group of similar patterns amounted to a mere .16. Because poor patterns are judged to having larger groups of similar patterns, the correlation means a weak tendency for poor patterns to possess larger complexity. The correlation between the ETC and judgments of figural goodness was similarly low at −.21. Good figures tend to be less complex. In sum, there exists an association by which subjectively poorer figures tend to be more complex in an objective sense. In this respect it is of interest to note that the correlation between the complexity algorithms of LZ and ETC over the same pool of patterns amounted to a significant .44. This is a decent correlation, yet it shows that different measures of complexity do not fully agree with one another (see Simon, 1972).

Could a third measure, palindrome complexity, better capture the objective underpinnings of figural goodness?

#### Applying Palindrome Complexity to Garner Stimuli

The third measure of complexity that we considered in the present effort was *subsymmetry* (Alexander & Carey, 1968). Subsymmetry is defined as a subset of neighboring elements within the stimulus that bear mirror symmetry (Alexander & Carey, 1968). Mirror subsymmetries are also called *palindromes,* and the total number of palindromes in a pattern is called its *palindrome complexity* (Anisiu et al., 2010). The more subsymmetries exist in a string, the more order and regularity it contains, and commensurably, the lower its palindrome complexity. To illustrate, the number of subsymmetries in the string 000 is 3 (i.e. 000, 00, 00) but the number of subsymmetries in the string 010 is 1 (i.e. 010). By palindrome complexity, the former string is less complex. In addition to their number, the length of subsymmetries is also important for figural goodness. To illustrate, the string 110011 contains two subsymmetries of length 2 (11 and 11), one subsymmetry of length 4 (1001) and one subsymmetry of length 6 (110011). This accounts for its perception as a good sequence/figure.

**Figure 6.**
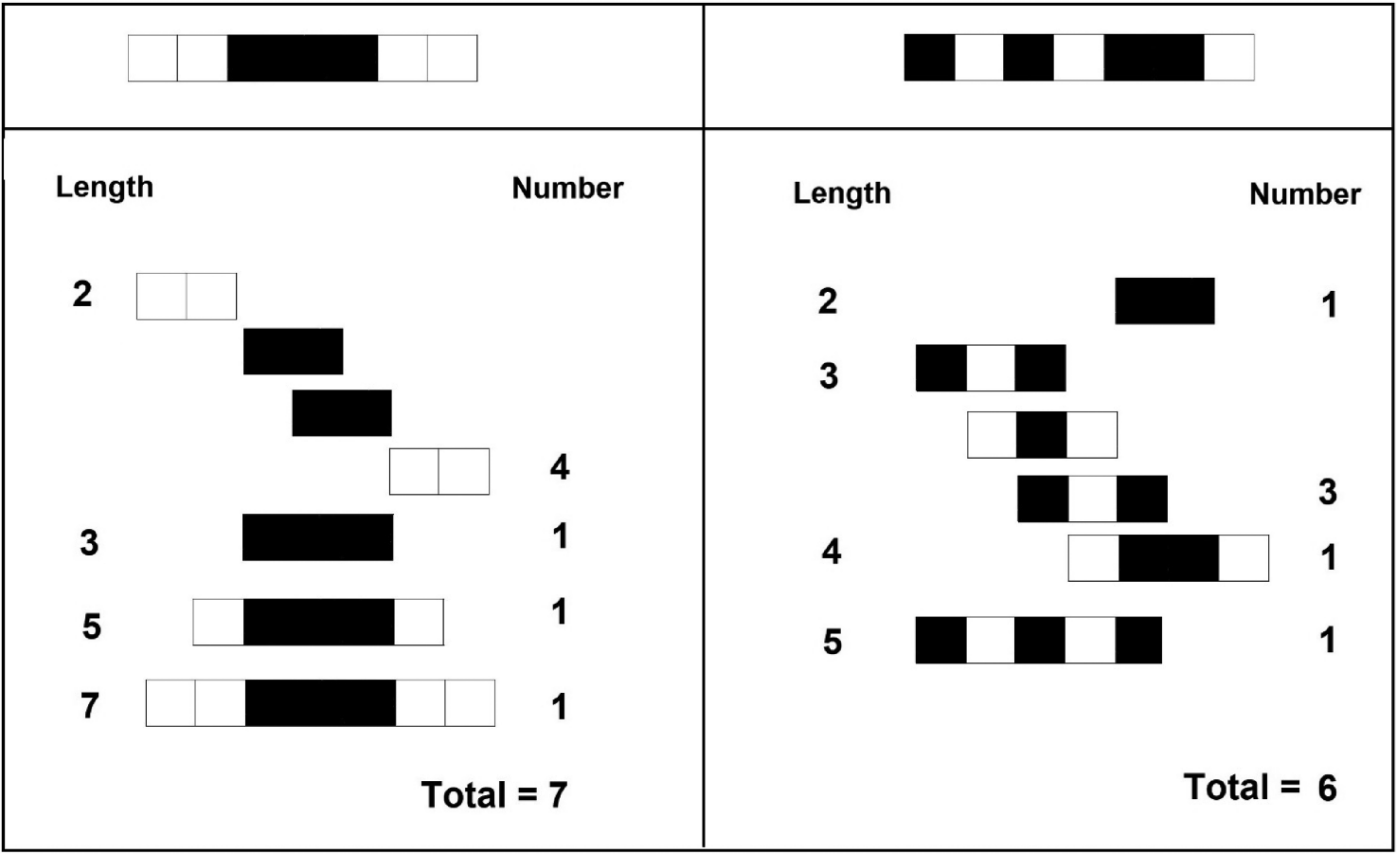
Top: Two unidimensional binary patterns. Beneath: Calculation of subsymmetries in each of the top patterns. Note that the total number of subsymmetries is larger in the pattern at the top left. Digit at the left: size of the subsymmetry; digit on the right: number of subsymmetries of that size. Note: Based on Toussaint et al. (2015).

Alexander and Carey (1968) counted the number of subsymmetries in one-dimensional binary patterns consisting of black and white squares and found that judgments of apparent complexity varied (inversely) with that number; the correlation between the number of subsymmetries and the apparent complexity amounted −.81. Clearly, a figure containing a high number of subsymmetries was considered simpler or less complex. This is also the case for the two sequences presented at the top of Figure 6: The figure on the left looks better than the figure on the right due perhaps to the greater number of subsymmetries that it contains.

Recently, Nagaraj and Balasubramanian (2017) defined a normalized measure of subsymmetries for unidimensional sequences of 0 and 1:

(4a) $$SubSym(x)1-\;\frac{subsymmetries(x)}{subsymmetries(Z)}$$

(4b) $$=1-\;\frac{subsymmetries(x)}{\left(\frac{n(n-1)}{2}\right)},$$

where *x* is the input sequence of length *n*, Z is the all-zero (or all-one) sequences of length *n*. The function computes the total number of subsymmetries. The all-zero sequence of length *n* has [*n*(*n *− 1)/2] subsymmetries, the largest number. The ratio in Equation 4a gives a measure of subsymmetry or palindrome complexity relative to Z. To provide a normalized measure, in Equation 4b this ratio is subtracted from 1. Thus, the resulting normalized measure is bounded between 0 (least complex) and 1 (most complex).

**Figure 7.**
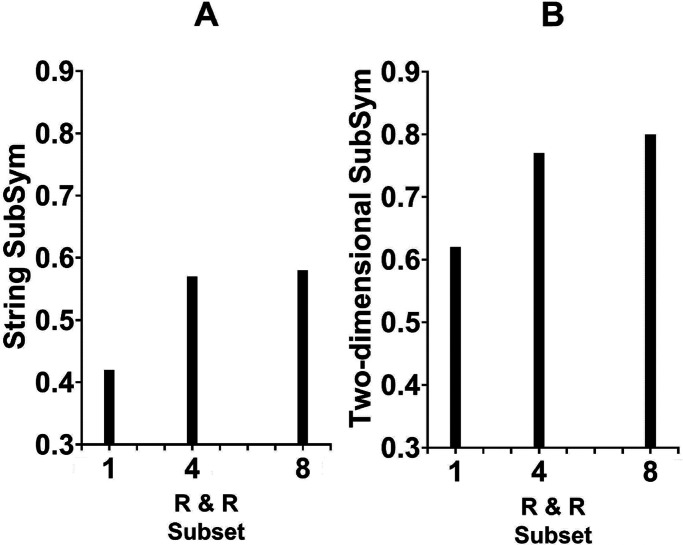
Panel a: Average unidimensional SubSym complexity as a function of the R & R subset. Panel b: Average two-dimensional SubSym complexity as a function of the R & R subset.

**Figure 8.**
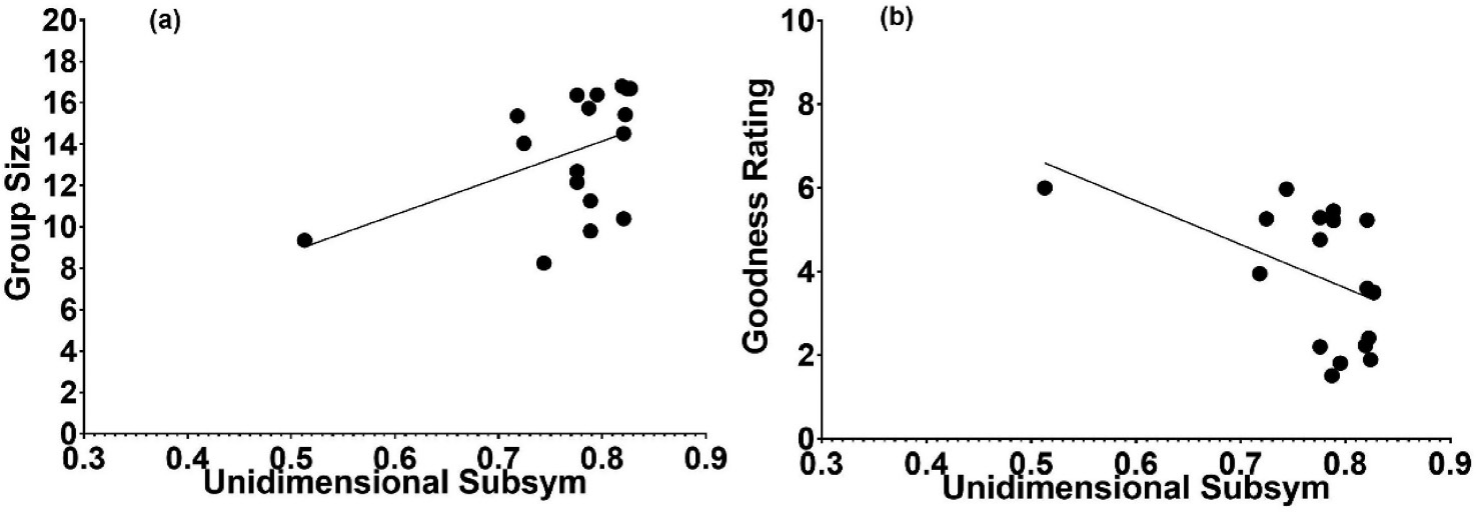
Two subjective variables from the Garner and Clement (1963) study plotted against the objective measure of the SubSym applied to the same stimuli. (a) Size of the subjective similarity group in which the stimulus exists as a function of the stimulus’ subsymmetry. (b) Judged figural goodness as a function of the figure's subsymmetry. Both contingencies are sizeable.

To relate the objective measures of SubSym (via Equations 4a and 4b) and R & R set size with respect to a given sequence, we followed the same routine used with LZ and ETC. The average SubSym values for all strings that belong to each R & R subset are presented in Figure 7a. The association is apparent but as is the case with respect to the LZ and ETC it is rather weak. Relating the subjective ratings from Garner and Clement (1963) to the current SubSym complexity yielded appreciable correlations (Figure 8a and b). The one between the SubSym and size of the judged similarity group was .42, indicating that poor strings were more complex by the objective measure of the SubSym. Significantly, the correlation between the judged figural goodness and the SubSym complexity was −.45. Strings standing for good figures were less complex by the SubSym. Could these correlations be produced by the outliers? To check, we calculated the pair of correlations without the outlier in each panel of Figure 8. For the relation between the group size and the SubSym at the left, deleting the outlier reduced the correlation into .17; for the relation between the rating of figural goodness and the SubSym at the right, deleting the outlier reduced the correlation into −.39. Both revised correlations were still marginally significant (*p*-values smaller than 0.1).

### Subsymmetry for Two-dimensional Spatial Patterns

**Figure 9.**
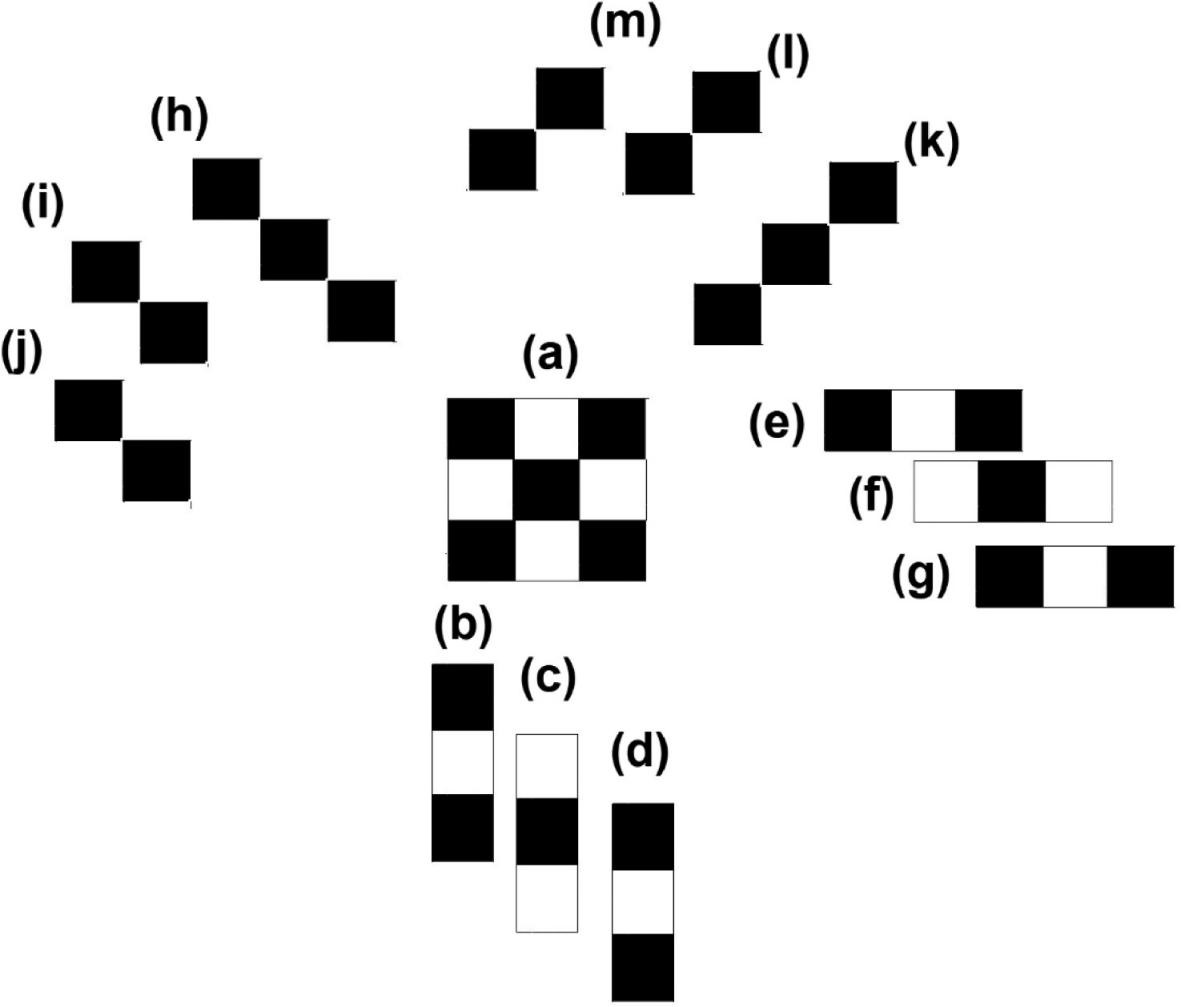
The subsymmetries of a square composed of small black and white squares (the two-dimensional pattern in the middle, (a); (b) horizontal subsymmetry of length 3; (c) horizontal subsymmetry of length 3; (d) horizontal subsymmetry of length 3; (e) vertical subsymmetry of length 3; (f) vertical subsymmetry of length 3; (g) vertical subsymmetry of length 3; (h) negative diagonal subsymmetry of length 3; (i) negative diagonal subsymmetry of length 2; (j) negative diagonal subsymmetry of length 2; (k) positive diagonal subsymmetry of length 3; (l) positive diagonal subsymmetry of length 2; and (m) positive diagonal subsymmetry of length 2.

Chipman (1977) extended the measure of the total number of subsymmetries into two-dimensional boards of black and white squares. She applied this measure successively to each of the rows and columns of the pattern, weighted by its length (say number of black squares). The correlation between the two-dimensional extension and subjective judgments of complexity amounted to .72. In the current contribution, we examined the subsymmetries of the Garner dot patterns. To illustrate, consider the square at the center of Figure 9 (pattern a) comprising small black and white squares. The patterns around the central target pattern (panels b–d, e–g, h–j, and m–k) illustrate the subsymmetries of that target pattern (Figure 9). Together, they specify the palindrome complexity of the central square.

Following Toussaint et al. (2015), we here developed a measure that takes into consideration the separate contributions of vertical subsymmetries (vSub), horizontal subsymmetries (hSub), right-diagonal (rdSub) and left-diagonal (ldSub) subsymmetries. Each type of subsymmetry is weighted by its string's length, L. The total subsymmetries (TotSub) is their sum, and therefore the measure amounts to

(5a) $$SubSym(x)=1-\frac{\sum_{i=1}^{n}\;\sum_{\;j=1}^{n}\;\sum_{k=1}^{k=2}\;L_{ijk}SubSym(x)}{\sum_{i=1}^{n}\;\sum_{\;j=1}^{n}\;\sum_{k=1}^{k=2}\;L_{ijk}SubSym(Z)}$$

(5b) $$=1-\frac{TotSub(x)}{TotSub(Z)},$$

where *Z* is an all-zero two-dimensional pattern, *i* is the number of rows, *j* the number of columns, *k* is the number of diagonals (2), and *L* is the length of the subsymmetry. Separate measures for only vertical or horizontal subsymmetries can also be constructed. Consider the two-dimensional square pattern in the middle of Figure 9. The total number of weighted subsymmetries is 32, whereas the comparable measure of the all-zero pattern is 56. Thus, the normalized measure of SubSym is 1–32/56 = 0.42. It is of note that this measure shares the logic of Zabrodsky Hel-Or's continuous measure of symmetry (Zabrodsky, 1993; Zabrodsky et al., 1993). The Zabrodsy Hel-Or development also measures overall symmetry as a distance from the perfect symmetry.

It is important to realize that this measure applies to the *spatial patterns* themselves rather than to (binary) sequences standing for them. We used Equations 5a and 5b to assess the two-dimensional SubSym complexity of the patterns themselves. Figure 7b shows the average values per R & R subset. The smaller the R & R set, the less complex are its members. Our spatial (two-dimensional) SubSym measure accounted quite well for the subjective ratings from Garner and Clement (1963).

**Figure 10.**
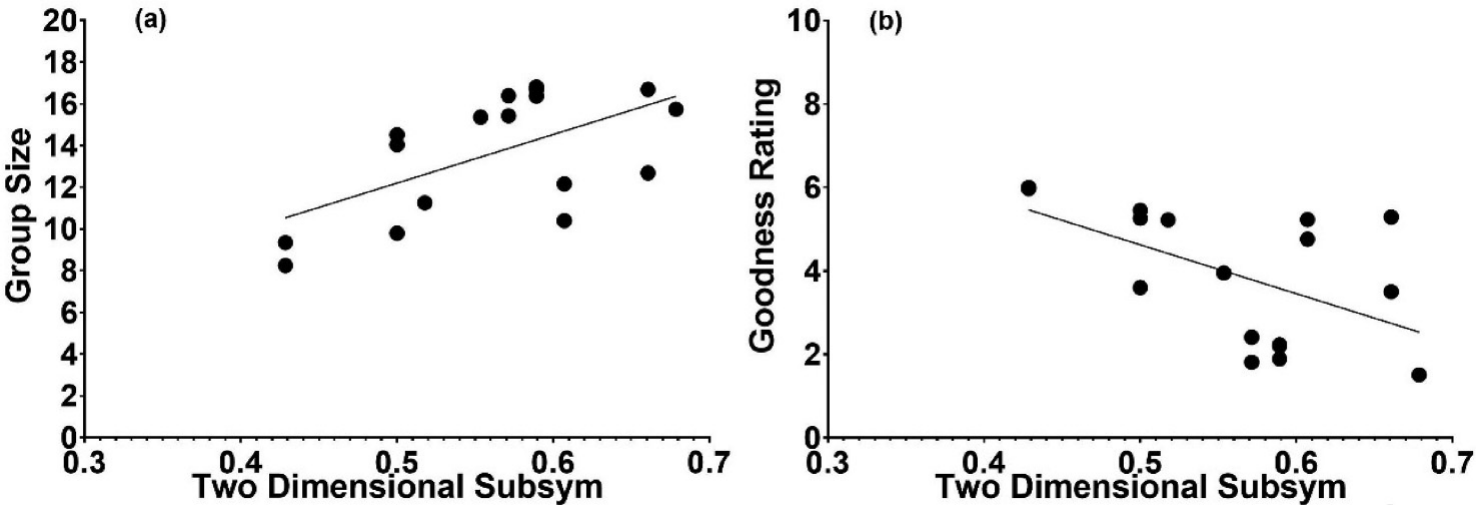
Two subjective variables from the Garner and Clement (1963) study plotted against the objective measure of two-dimensional SubSym applied to the same stimuli. (a) Size of subjective similarity group in which the stimulus exists as a function of the two-dimensional stimulus subsymmetry. (b) Judged figural goodness as a function of the figure's two-dimensional subsymmetry. Both contingencies are sizeable.

We also related the subjective ratings from Garner and Clement (1963) to the current two-dimensional SubSym complexity measure (Figure 10a and b). The correlation between the SubSym and size of the judged similarity group was .59, indicating that subjectively poor strings were more complex by the objective measure of the two-dimensional SubSym. Also significant was the correlation between the judged figural goodness and the two-dimensional SubSym complexity, the value amounting to −.54. Perceptually good figures are less complex as measured by the two-dimensional SubSym.

We should add an important caveat with respect to the correlations reported in our novel analyses. They are meant as descriptive measures to provide a sense of the associations. The measures are constrained by the fact that we used data collected by various investigators (i.e. not our own data). As well, the measures are constrained by the nature of the stimuli tested and the properties of the algorithmic routines developed.

## The Mathematics–Psychology Chasm: Graded Surrogates for Perceptual Symmetry

**Figure 11.**
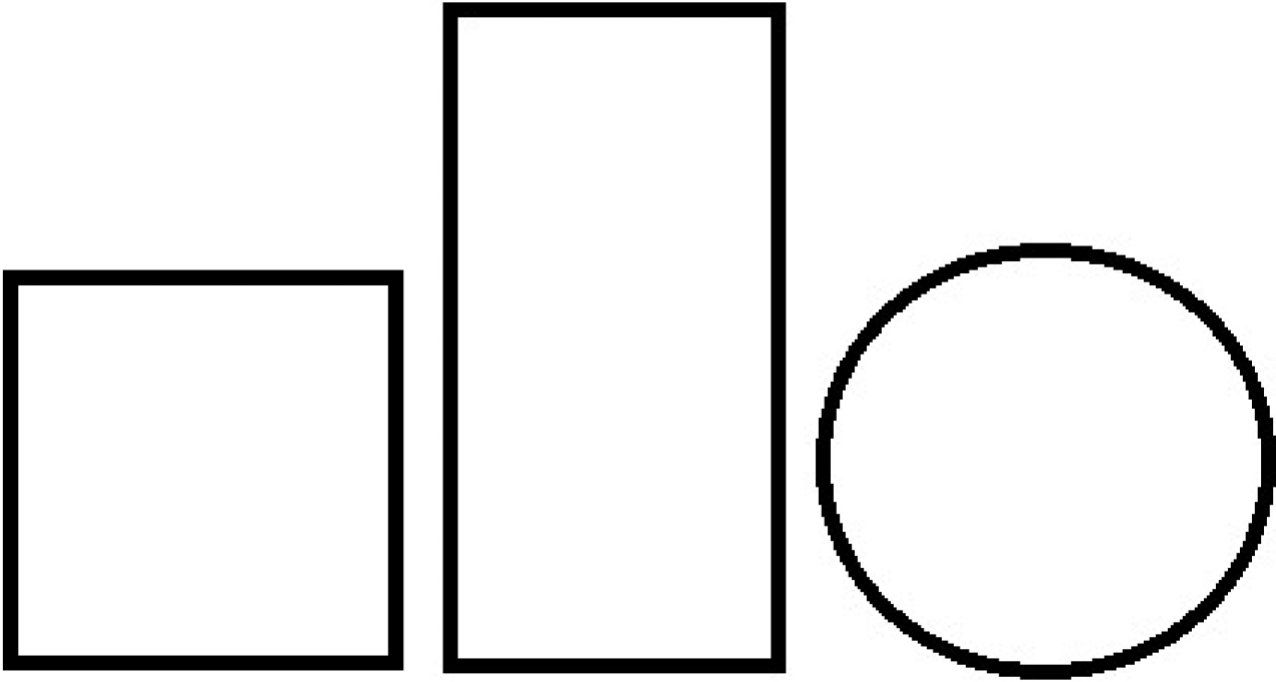
The mathematics–psychology chasm. When asked which figure, the square or the rectangle, is more symmetrical, most participants do not understand the question and when they do provide an answer it is typically the wrong one. Most participants respond that the quality of symmetry does not apply to a circle.

The naïve binary conception of symmetry—an object is either symmetrical or is nonsymmetrical—governs laboratory and popular parlance (Zabrodsky & Algom, 1994). In that respect, we note the increasingly sophisticated literature on symmetry detection (e.g. Baylis & Driver, 2001, 1995; Bertamini et al., 1997, 2018; see also, Treder, 2010). A range of visual regularities, including various symmetries, have been documented along with the attendant neural processing systems. The valuable contributions notwithstanding, the very term “symmetry detection” supports, if unwittingly, the naïve dichotomy, symmetric versus nonsymmetric. The dichotomy is groundless however, because in mathematics, symmetry is a *graded* variable, not a discrete dichotomous (present/absent) one. Thus various objects entail different amounts of symmetry. This property is not captured in large portions of symmetry studies in psychology. Psychologists have not yet revealed subjective analogs to key concepts of symmetry within mathematical group theory including those of the symmetry group, size of symmetry group, type and number of symmetries, or the structure of the symmetry group (as conveyed for example by Cayley diagrams). Given the binary conception, asking people to assess the symmetry of the presented stimulus seemed worthless to investigators (Figure 11).

Again, at the input end, symmetry is a fine-grained independent variable, which, at the conceptual level, is incommensurate with a dichotomous dependent variable. Unlike the dichotomous subjective symmetry, the perceptual experience of figural goodness *is* a graded variable that people find easy to apply to stimuli. Therefore, figural goodness increasingly served as a surrogate for “symmetry-sensation” (generated by physical symmetry). In time, the functional relations between symmetry and further *graded* sensations were explored, chief among the latter being subjective randomness and subjective complexity.

Although people find the dichotomy, random–nonrandom, almost as compelling as the dichotomy, symmetrical–nonsymmetrical, the former still proved amenable to meaningful graded ratings (Falk, 1975; Falk & Konold, 1997; Lopes & Oden, 1987). Apparent randomness thus gained traction as a popular dependent variable (ignoring the fact that randomness lacks an exact consensual definition; Lopes, 1982). For students of symmetry, apparent randomness proved attractive in their quest to uncover the perceptual effects of symmetry. A completely random stimulus (e.g. an unpredictable binary string) lacks symmetry and patterness; conversely, nonrandom stimuli contain symmetry and patterness. Consequently, symmetry has been incorporated into formal models of apparent randomness (e.g. Griffiths & Tenenbaum, 2003; Lopes & Oden, 1987). The “difficulty predictor” (DP) model developed by Falk and Konold (1997) is based on the relative frequency of runs (subsequences containing the same symbol) in the stimulus. Note that such subsequences entail symmetry by definition, an idea captured in the notion of subsymmetry that we discussed. Random and nonrandom stimuli specify two extreme boundary conditions, the inverse of nonsymmetry and symmetry, respectively. An open question concerns the granularity of this relation, the relation between randomness and patterness throughout a range of values.

A third graded variable, both at the stimulus and the sensation ends, is complexity, a remote tentative surrogate for symmetry. The concept entails the analysis of stimuli (say, strings) by the length of the computer program needed to generate them. The programs take advantage of any patterns lurking in the string so that they are (much) shorter than the stimulus string that they reproduce. Patternless random strings, by contrast, require programs that are as long as the stimulus string itself. In other words, patterned stimuli are less complex, hence compressible, whereas random stimuli are noncompressible. We also note that people find *subjective* complexity easy to rate, and this experiential variable was often recorded well before the recent wave of algorithmic complexity. Complexity thus evolved into the linchpin of contemporary research in the domain of randomness and patterness, and, of necessity, of symmetry.

However, does the presence of patterns mean the presence of good patterns? Recall that, in lieu of rated subjective symmetry, researchers have been using figural goodness as an assay for the perceptual effect. To repeat, nonrandom stimuli imply patterns in the stimuli; the question is, do they imply that the patterns are good patterns? Algorithmic complexity might be too coarse to capture figural goodness and other cognitive functions like learning and discrimination (Griffiths & Tenenbaum, 2003). For illustration, consider the pair of strings below:

$$\begin{array}{rl} & 1\;1\;1\;1\;1\;1\\ & 1\;2\;2\;3\;3\;3 \end{array}$$

Both strings have six digits, and both appear nonrandom, yet they look different with the bottom one more complex. However, many computer programs of complexity yield the same outcome: two instructions suffice to generate each sequence. For the top: start with *n* = 1 and then repeat it six times; for the bottom: for *n* = 1 to 3, repeat *n n* times (inspired by Lopes, 1982).

Again, algorithmic complexity may not capture the finesse of human perception. Fine tuning of the relation between complexity, algorithmic and figural goodness, and symmetry is a task for future studies.

## Conclusion

We attempted to redress what we perceived to be an imbalance in the literature by focusing on the conceptual and mathematical roots of the symmetry. In that context, we reviewed several early and a sample of modern approaches. Notably, an attempt was made to conjoin measures from hitherto separate research. For example, we related algorithmic complexity and figural goodness (as defined by inferred set size). We revealed an association as we did with further measures of complexity and figural goodness. However, this early foray also revealed the limitations posed by existing tools.

Integrating the various approaches to symmetry remains a daunting task for cognitive and mathematical psychologists.
